# Bilateral Breast Reconstruction Using Extended Latissimus Dorsi Musculocutaneous Flaps for Metachronous Bilateral Breast Cancer: A Case Report

**DOI:** 10.7759/cureus.88024

**Published:** 2025-07-15

**Authors:** Natsuno Iwaisako, Shoji Oura

**Affiliations:** 1 Department of Surgery, Kishiwada Tokushukai Hospital, Kishiwada, JPN

**Keywords:** bilateral breast reconstruction, breast cancer, cosmetic outcomes, latissimus dorsi musculocutaneous flap, respiratory function

## Abstract

A 49-year-old woman had undergone radiofrequency ablation (RFA) therapy and sentinel lymph node biopsy (SNB), followed by radiation therapy for her right breast cancer at the age of 31. The patient had further undergone nipple-sparing mastectomy (NSM), SNB, and immediate breast reconstruction using an extended latissimus dorsi musculocutaneous flap (eLDMCF) for her left breast cancer at the age of 43. Follow-up mammography further revealed widespread linear calcifications in the right breast. Core needle biopsy pathologically showed atypical cells growing in trabecular and tubular fashions with connective tissue proliferation, leading to the diagnosis of invasive ductal carcinoma. Due to the patient's strong preference for not using silicone prosthesis on right breast reconstruction, the patient underwent NSM and SNB, followed by immediate breast reconstruction using the right eLDMCF after obtaining full informed consent about the unknown bilateral eLDMCF harvesting effect on respiratory function. The patient recovered uneventfully and showed respiratory function as follows: preoperative 2.69 L to postoperative 2.46 L in vital capacity and preoperative 2.1 L to postoperative 1.83 L in forced expiratory volume in one second. The patient reported no respiratory symptoms and has been fully satisfied with the cosmetic outcomes of the reconstructed right breast. These results suggest that bilateral breast reconstruction using eLDMCFs can be a good therapeutic option for metachronous bilateral breast cancer.

## Introduction

In the treatment of breast cancer, cure is undoubtedly the main goal, while postoperative cosmetic outcomes are another important matter. Therefore, patients' strong request for favorable cosmetic outcomes has made breast-conserving therapy one of the standard therapeutic options for early breast cancer [1]. However, various breast cancer-related factors such as tumor size and multicentricity have prevented many breast cancer patients from benefiting from breast-conserving therapy. Breast reconstruction, therefore, has been of great benefit to many breast cancer patients with exclusion factors for breast-conserving therapy [2].

Plastic surgeons use artificial materials [3], autologous tissues [4], or both [5] in breast reconstruction. They have exclusively used artificial materials in bilateral breast reconstruction for synchronous bilateral breast cancer [6]. Autologous tissues can be used in breast reconstruction for metachronous breast cancer, i.e., two cancers occurring more than two months apart, but plastic surgeons should pay much attention to the complications after harvesting the recipient tissue.

Bilateral breast reconstruction using bilateral extended latissimus dorsi musculocutaneous flaps (eLDMCFs) (which harvest the latissimus dorsi and overlying subcutaneous fat up to the iliac crest) inevitably necessitates multiple positional changes, consequently requires prolonged operation time, and has never been a common option for bilateral breast reconstruction of synchronous bilateral breast cancer. In addition, it remains unclear to what extent harvesting the bilateral eLDMCFs affects respiratory function in patients with synchronous/metachronous bilateral breast cancer. In short, due to the relative rarity of bilateral breast reconstruction using autologous tissues, to date, limited studies have reported the clinical outcomes, including respiratory function, of bilateral breast reconstruction using bilateral eLDMCFs, even in cases of metachronous bilateral breast cancer.

We, herein, report a case in which breast reconstruction using bilateral eLDMCFs for metachronous bilateral breast cancer resulted in favorable cosmetic outcomes without any side effects, including significant deterioration of respiratory function.

## Case presentation

**Figure 1:**
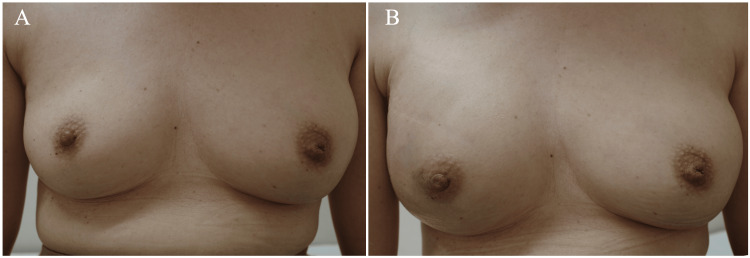
Cosmetic outcomes A: The reconstructed left breast using an eLDMCF was slightly larger than the right breast after radiofrequency ablation and radiotherapy. B: The right breast after the eLDMCF breast reconstruction became larger than that before breast reconstruction. eLDMCF: extended latissimus dorsi musculocutaneous flap

A 49-year-old woman had undergone radiofrequency ablation (RFA) therapy [7] and sentinel lymph node biopsy (SNB), followed by adjuvant radiation therapy (50 Gy) for her right breast cancer at the age of 31. Due to estrogen and progesterone receptor negativity, the patient received four cycles of anthracycline-containing chemotherapy. The patient had further undergone nipple-sparing mastectomy (NSM) and SNB, followed by immediate breast reconstruction using an eLDMCF for her left breast cancer, which was invasive ductal carcinoma and had both estrogen and progesterone receptor positivity (both Allred score 8), human epidermal growth factor receptors type 2 negativity, and a high Ki-67 labeling index of 80%. Due to the extremely high Ki-67 labeling index, the patient received four cycles of docetaxel and cyclophosphamide chemotherapy, and thereafter, exemestane and a luteinizing hormone-releasing hormone analogue. The reconstructed left breast using the eLDMCF had shown excellent cosmetic outcomes for more than five years and had been slightly larger than the right breast treated with RFA and adjuvant radiotherapy (Figure 1A).

**Figure 2:**
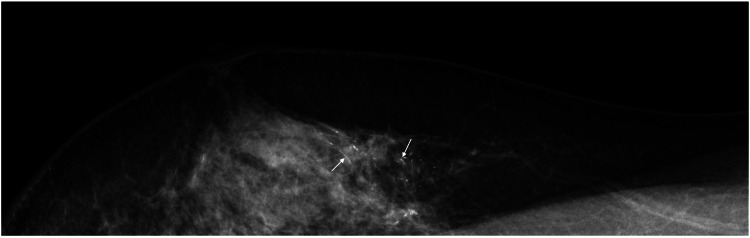
Mammography findings Mediolateral oblique view mammography showing widespread linear calcifications (arrows) in the upper part of the right breast.

Annual mammography further revealed widespread linear microcalcification in the right breast 18 years after the right breast cancer operation (Figure 2).

**Figure 3:**
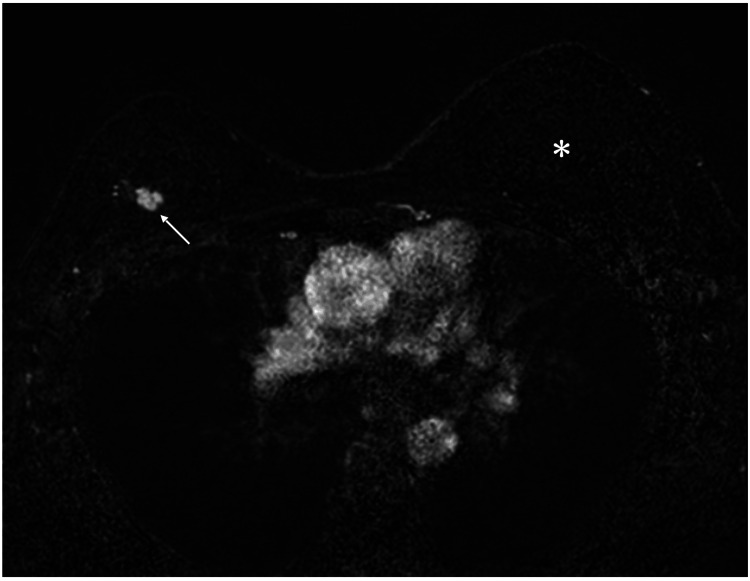
MRI findings MRI showed that the right breast was smaller than the reconstructed left breast (asterisk) after radiofrequency ablation and radiotherapy, in addition to the fast enhancement (arrow) of the right breast cancer. MRI: magnetic resonance imaging

Ultrasound showed an irregular mass, 11 mm in size, in the right upper outer quadrant of the breast and a presumed RFA-induced cystic lesion in the right upper inner quadrant of the breast (figures not shown). Magnetic resonance imaging of the right breast showed fast enhancement in the initial phase and a smaller breast volume compared to that of her reconstructed left breast (Figure 3).

**Figure 4:**
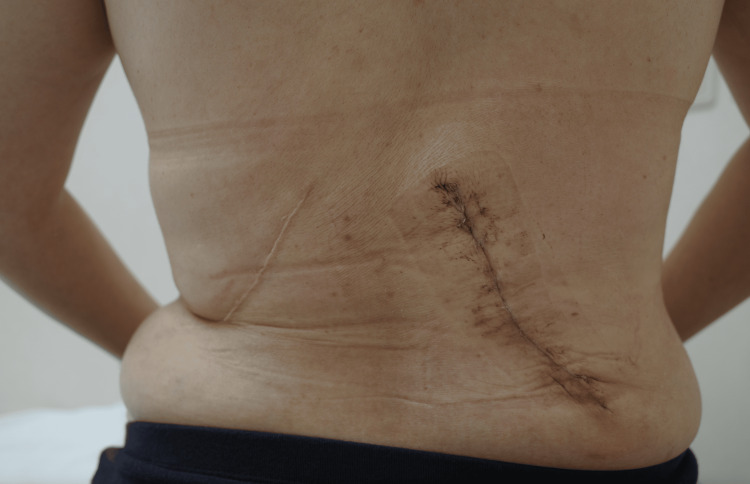
Extent of extended latissimus dorsi musculocutaneous flap harvesting Appearance of the back just after discharge showing bilateral oblique skin scars, which were quite different from those of the standard latissimus dorsi musculocutaneous flap breast reconstruction.

Under the presumed diagnosis of breast cancer, core needle biopsy was performed on the right breast mass, leading to the diagnosis of triple-negative breast cancer with a Ki-67 labeling index of 22%. The right breast, irradiated after the RFA and SNB, had extensive cancer spread, leading to the judgment of impossible application of breast-conserving therapy for the presumed newly developed right breast cancer. The patient requested us to reconstruct her right breast again using the eLDMCF in her right breast cancer operation. The patient, therefore, underwent NSM and SNB, followed by immediate breast reconstruction using the eLDMCF (Figure 4) and showed excellent cosmetic outcomes of the reconstructed right breast (Figure 1B).

The patient recovered uneventfully, was discharged on the 14th day after operation, and showed respiratory functions as follows: preoperative 2.69 L to postoperative 2.46 L in vital capacity and preoperative 2.1 L to postoperative 1.83 L in forced expiratory volume in one second (Table 1). Postoperative pathological study showed that the right breast cancer had a triple-negative phenotype, maximal invasive size of 12 mm, extensive ductal spread, and a Ki-67 labeling index of 18%. Due to the prior exposure to anthracycline and taxane agents, the patient started to receive oral TS-1 chemotherapy and is scheduled to be followed on an outpatient basis.

**Table 1:** Respiratory function ERV: expiratory reserve volume, IRV: inspiratory reserve volume, FVC: forced vital capacity, MMF: maximum mid‑expiratory flow rate

Respiratory function variables	Before first operation	Before second operation (156 months later)	Before third operation (224 months later)	2 weeks after third operation
Vital capacity (L)	3.69	3.06	2.69	2.46
Tidal volume (L)	0.89	0.84	0.61	0.73
ERV (L)	1.44	1.07	0.81	0.71
IRV (L)	1.35	1.16	1.27	1.02
FVC (L)	3.59	3.13	2.69	2.40
FEV1.0 (L)	2.98	2.47	2.10	1.83
FEV1.0%	83.0	78.9	78.1	74.4
MMF (L)	2.93	2.16	1.84	1.46

## Discussion

Generally, plastic surgeons reconstruct bilateral breasts using silicone implants for simultaneous bilateral breast cancer ineligible for breast-conserving therapy [6]. The risk of developing malignant lymphoma [8], however, has significantly reduced the frequency of silicone implant use not only in bilateral breast reconstruction but also in unilateral breast reconstruction. Despite being fully informed of the low incidence of silicone implant-related malignant lymphoma, the patient firmly refused to use a silicone implant for her right breast reconstruction.

Compared to the rectus abdominis musculocutaneous flap, the latissimus dorsi musculocutaneous flap (LDMCF) has the limitation of breast reconstruction volume. The LDMCF, therefore, is most frequently used for cosmetic improvement on large lumpectomies [9].

The LDMCF has the advantage of stable blood flow and is therefore unlikely to develop major flap necrosis. Many researchers, including us, therefore, efficiently use LDMCF as an eLDMCF to further improve cosmetic outcomes of the reconstructed breast after NSM. Although radiation therapy and aging for over 18 years might have made the right breast slightly smaller, the reconstructed left breast using the eLDMCF showed a slightly larger volume than the irradiated right breast after RFA and showed excellent cosmetic outcomes.

The latissimus dorsi muscle has extensive attachments to the thorax and is known as an accessory muscle of respiratory function [10]. It is generally understood that its impact on respiratory function is clearly less than that of the diaphragm and intercostal muscles [11]. However, the effect of latissimus dorsi muscle function loss, especially of bilateral latissimus dorsi muscle harvesting, on respiratory function has not yet been fully investigated to date. In fact, we evaluated the patient's respiratory function before and after the metachronous bilateral breast reconstruction using bilateral eLDMCFs and found some decline in respiratory function, but no perceivable negative effects on breathing and daily life of the patient. Therefore, in patients with normal respiratory function, the use of bilateral eLDMCFs for bilateral breast reconstruction can give great benefit to metachronous bilateral breast cancer patients without any clinically detectable major side effects.

Breast specialists should clarify which patients can get maximum benefit from breast reconstruction using the eLDMCF. The eLDMCF reconstruction, however, should be an excellent reconstructive option for patients with not very large breasts. Furthermore, breast surgeons should note that in healthy breast cancer patients with normal respiratory function, the use of bilateral eLDMCFs for bilateral breast reconstruction in cases of metachronous bilateral breast cancer is an excellent therapeutic option.

## Conclusions

Harvesting of eLDMCF affects respiratory function, but only to a minor extent. The use of LDMCFs in breast reconstruction for synchronous bilateral breast cancer is impractical due to the long operative time required. Breast surgeons, however, should note that in the reconstruction of metachronous bilateral breast cancer, the use of eLDMCFs can be of much benefit to patients without respiratory function abnormalities.
